# Development of a Lyophilization Process for *Campylobacter* Bacteriophage Storage and Transport

**DOI:** 10.3390/microorganisms8020282

**Published:** 2020-02-19

**Authors:** Lu Liang, Nicholas B. Carrigy, Samuel Kariuki, Peter Muturi, Robert Onsare, Tobi Nagel, Reinhard Vehring, Phillippa L. Connerton, Ian F. Connerton

**Affiliations:** 1 Division of Microbiology, Brewing and Biotechnology, School of Biosciences, University of Nottingham, Sutton Bonington Campus, Loughborough LE12 5RD, UK; sbzll3@nottingham.ac.uk (L.L.); sbzplc@nottingham.ac.uk (P.L.C.); 2 Department of Mechanical Engineering, University of Alberta, Edmonton, AB T6G 1H9, Canada; carrigy@ualberta.ca (N.B.C.); reinhard.vehring@ualberta.ca (R.V.); 3 Center for Microbiology Research, Kenyan Medical Research Institute, 54840 Nairobi, Kenya; samkariuki2@gmail.com (S.K.); pmutury@yahoo.com (P.M.); robert.s.onsare@gmail.com (R.O.); 4 Phages for Global Health, Oakland, CA 94618, USA; tobi@phagesforglobalhealth.org

**Keywords:** *Campylobacter*, bacteriophage, lyophilization, phage transportation, phage stability

## Abstract

Bacteriophages are a sustainable alternative to control pathogenic bacteria in the post-antibiotic era. Despite promising reports, there are still obstacles to phage use, notably titer stability and transport-associated expenses for applications in food and agriculture. In this study, we have developed a lyophilization approach to maintain phage titers, ensure efficacy and reduce transport costs of *Campylobacter* bacteriophages. Lyophilization methods were adopted with various excipients to enhance stabilization in combination with packaging options for international transport. Lyophilization of *Eucampyvirinae* CP30A using tryptone formed a cake that limited processing titer reduction to 0.35 ± 0.09 log_10_ PFU mL^−1^. Transmission electron microscopy revealed the initial titer reduction was associated with capsid collapse of a subpopulation. Freeze-dried phages were generally stable under refrigerated vacuum conditions and showed no significant titer changes over 3 months incubation at 4 °C (*p* = 0.29). Reduced stability was observed for lyophilized phages that were incubated either at 30 °C under vacuum or at 4 °C at 70% or 90% relative humidity. Refrigerated international transport and rehydration of the cake resulted in a total phage titer reduction of 0.81 ± 0.44 log_10_ PFU mL^−1^. A significantly higher titer loss was observed for phages that were not refrigerated during transport (2.03 ± 0.32 log_10_ PFU mL^−1^). We propose that lyophilization offers a convenient method to preserve and transport *Campylobacter* phages, with minimal titer reduction after the drying process.

## 1. Introduction

*Campylobacter* is the leading cause of foodborne zoonotic gastrointestinal disease [1]. According to the Centers for Disease Control and Prevention, *Campylobacter* has been identified as the most common form of foodborne infection since 2013 in the USA and caused the highest number of infections (19.5 cases per 100,000 population) during 2018, representing a 12% increase in incidence compared to 2015–2017 [2]. Among the reported cases, 18% of infected people were hospitalized (1811) and 0.3% died (30). Poultry is the most common reservoir for *Campylobacter* spp. National Antimicrobial Resistance Monitoring System for Enteric Bacteria (NARMS) reported 24% of sampled retail chickens were *Campylobacter* positive with 17.9% isolates resistant to ciprofloxacin [3]. The US Department of Agriculture’s Food Safety and Inspection Service (USDA-FSIS) isolated *Campylobacter* from 18% of sampled chicken carcasses and 16% of sampled chicken parts [4]. In the UK, Public Health England reported that *Campylobacter* caused 56,729 cases of infection (96.57 cases per 100,000 population) in 2017 and that 56% of the sampled retail whole chicken were *Campylobacter* contaminated [5,6].

In developing countries, the surveillance systems for *Campylobacter* are often incomplete or absent due to lack of resources (e.g., trained scientists, funding, infrastructure) compared with the developed world. The disease burden of *Campylobacter* in low- and middle-income countries is therefore either underestimated or unknown. In Kenya’s capital Nairobi, *Campylobacter* is prevalent, with 33%–44% of broiler chicken from small farms and 60–64% retail chickens being positive [7]. *Campylobacter* infection in Kenya has the world’s highest fatality rate of 8.8% (5/57) within children under 5 years old in hospitals [8].

There is no doubt that the prevalence of *Campylobacter* in both developed and developing countries needs to be limited, and in response to this the European Union has placed a limit of 1000 CFU/g on broiler chicken carcasses (Commission Regulation (EU) 2017/1495). Researchers have shown promising results using phages to reduce the level of *Campylobacter* on chicken carcasses [9,10,11,12], and in vivo without affecting the host microbiota structure [13,14,15,16,17]. We believe phage treatment is particularly suitable for applications in developing countries since phage products are relatively low-cost and robust and can be safely handled and incorporated into local good practice guidelines for farm and slaughterhouse use. Prebiotics and probiotics have been investigated as sustainable interventions to reduce *Campylobacter* levels in host animals through intestinal competition [18,19,20,21]. These approaches may be complementary to phage applications on farms. However, phages can also be applied as a directed bio-sanitation tool against *Campylobacter* that can penetrate and disrupt biofilms to kill bacteria [22].

To realize the promise of phage therapy to reduce the burden of pathogenic bacteria, studies have focused on making dry powder preparations for phages to maintain titer stability, extend product shelf-life and transport range, provide ease of handling and, optionally, to enable application as an inhalable product [23,24,25,26]. There are two common drying processes: lyophilization, which removes ice from frozen samples by sublimation under vacuum, and spray-drying, which evaporates water from an atomized liquid sample in a hot drying gas. In one study [24], lyophilization caused less damage to phages than spray-drying, and it has been widely applied in biopharmaceutical industries to produce therapeutic proteins and vaccines. After lyophilization, the product confers stabilization via solid glass formation and possesses a decreased molecular mobility, with less chance to encounter microbial contamination, although Wang has pointed out there remain many routes for physical and chemical instability [27]. In this study, we have focused on designing a systematic lyophilization method to produce freeze-dried *Campylobacter* bacteriophage CP30A suitable for transport, enabling phage therapy candidates to be screened and propagated in the pursuit of the international development of sustainable phage products to reduce the global disease burden.

## 2. Materials and Methods

### 2.1. Strains and Culturing Conditions

Bacteriophage CP30A (*Eucampyvirinae* Fletchervirus) was used in this study, which represents an isolate recovered from poultry excreta [13]. We used *Campylobacter jejuni* PT14 (NCBI accession CP003871) [28] as the host bacteria to propagate phage CP30A. *C. jejuni* strains PT1, HPC5, TIVC9, GH2F7, BIIIC3, HPIF9, F2E3, F2C10, NCTC 11168 and *C. coli* PT44 were used to confirm the host range of the treated bacteriophage CP30A [29]. Blood agar (BA) base No.2 plates (Oxoid, Basingstoke, UK) supplemented with 5% (*v/v*) defibrinated horse blood (TCS biosciences, Buckingham, UK) was used for routine bacteria culturing. The host bacterium was grown under microaerobic conditions (5% *v/v* oxygen, 2% *v/v* hydrogen, 88% *v/v* nitrogen, 5% *v/v* carbon dioxide) in a Whitley M35 Variable Atmosphere Workstation (Don Whitley Scientific, Bingley, UK) at 42 °C for 18 hours.

### 2.2. Phage Propagation and Titration

A modification of a whole plate lysis method was adapted to propagate phage from that described previously [30]. In brief, overnight-grown bacteria on BA plates were harvested in 10 mL of 10 mM magnesium sulfate solution. Five hundred microliter aliquots of bacterial suspension were mixed with 100 µL of phage at a titer of approximately 7 log_10_ PFU mL^−1^. This *Campylobacter*/phage mix was then added to 5 mL of molten NZCYM top agar and poured onto a NZCYM plate. The plates were left to dry and incubated at 42 °C under microaerobic conditions for 18 hours. For phage harvest, 5 mL of reverse osmosis (RO) water was transferred onto the plate and incubated at 4 °C with shaking overnight. After incubation, the recovered phage lysate was filtered through a 0.22 µm filter to remove bacteria.

To titer the propagated phage, a bacterial lawn was firstly prepared by harvesting overnight-grown bacteria in 10 mL of 10 mM magnesium sulfate solution, followed by mixing 500 µL of bacterial suspension aliquot with 5 mL of molten NZCYM top agar and pouring onto NZCYM plate. The plates were left to dry and 10 µL of 10-fold serial diluted phage samples in SM buffer (50 mM Tris.HCl (pH7.5), 100 mM NaCl, 8 mM MgSO4, 0.01% Gelatin) were then spotted onto bacterial lawn in triplicate. Once the spots were dried, the plates were incubated at 42 °C under microaerobic conditions for 18 hours. The resulting plaques were counted, and the titer determined as the plaque forming unit (PFU) per mL.

### 2.3. Phage Buffer Exchange

Prior to application of any drying approaches, phage CP30A was buffer-exchanged to reverse osmosis water in order to remove any impurities and media ingredient carry over unless otherwise stated. This was achieved by centrifugation or ultrafiltration. In brief, the filtered phage CP30A was pelleted by centrifuging at 4 °C with 39,000 × *g* for 2 hours. After centrifugation, the supernatant was removed, and the pellet was resuspended in reverse osmosis water with a volume equivalent to the applied phage lysate. In parallel, a Vivaspin 20 column with molecular weight cut off (MWCO) of 100 kDa (Merck, Cramlington, UK) was also applied to purify phages. To determine the concentration of solid matter, 1 mL of phage CP30A was spotted onto the surface of a pre-weighed petri dish. After air-drying in a ventilated hood, the weight increase of the petri dish was recorded as solid matter concentration.

### 2.4. Lyophilization

One milliliter of phage CP30A sample with a titer of approximately 9 log PFU mL^−1^ was frozen at −80 °C for 30 minutes in a 10 mL freeze-drying vial and subsequently lyophilized in a Christ Alpha 1-2 LDplus freeze dryer (Christ, Osterode, Germany) at −65 °C under 0.013 mbar pressure for 24 hours. After lyophilization, samples were sealed in the vial to maintain vacuum. Three biological replicates were applied for each formulation. To rehydrate, 1 mL of reverse osmosis water was added to the lyophilized phage-cake followed by gentle shaking.

### 2.5. Moisture Content Measurement

The moisture content of lyophilized phage CP30A was measured using a Thermogravimetric Analyzer TGA/DSC 3+ (Mettler-Toledo, Leicester, UK) according to manufacturer’s instruction. In brief, 2.7 mg of lyophilized phage CP30A was loaded onto TGA empty pan and heated from 23 °C to 550 °C at a ramp rate of 0.167 °C s^−1^. Samples were weighed every 4 s, and the moisture content was calculated from the change in mass by the instrument after the run.

### 2.6. Intertional Transportation

Lyophilized phage CP30A was shipped from Nottingham, UK to Nairobi, Kenya either at ambient temperatures or by refrigerated delivery services provided by Carramore International Ltd (Holmfirth, UK). The shipments contained three biological replicates for each formulation. An identical set of samples was prepared from the same batch and incubated in the Nottingham laboratory either at 4 °C or 30 °C as controls. Lyophilized samples arrived in Kenya 23 days post-shipment. They were rehydrated upon arrival and the phage titer was enumerated. Control samples were rehydrated and enumerated at the same time in the Nottingham laboratory. Both sets of rehydrated samples were also titered one week after rehydration to determine the stability of the recovered phages.

### 2.7. Scanning Electron Microscopy and Transmission Electron Microscopy

After incubating at 4 °C or 30 °C under vacuum for 23 days in the Nottingham laboratory, the lyophilized phage CP30A was examined with Jeol JSM-6060LV Scanning Electron Microscope (Jeol, Herts, UK) together with freshly prepared lyophilized samples as controls. The lyophilized phage CP30A was gently transferred onto 12.5 mm Scanning Electron Microscopy (SEM) pin stubs and coated with platinum via a Polaron SC7640 sputter coater (Quorum Technologies, Kent, UK) followed by SEM.

Once the lyophilized phage CP30A was rehydrated, they were examined with an FEI Tecnai G2 12 Biotwin Transmission Electron Microscope (ThermoFisher, Leicestershire, UK). Firstly, 14 µL of phage suspension was loaded onto a formvar/carbon film on copper 200 mesh grid (EM resolution, Sheffield, UK) and left for 3 minutes. The suspension was removed with lens paper, and 14 µL of 0.5% *w/v* uranyl acetate was added onto the grids to negative stain the phage for one minute. After staining, uranyl acetate was removed with lens paper and the grid was examined by transmission electron microscopy (TEM).

### 2.8. Statistical Analysis

Titer loss of phages postlyophilization was presented as the mean plus or minus the standard deviation of log_10_-transformed PFU mL^−1^ determined from three biological replicates. Statistical significance was calculated using the *t*-test at a significance level of 0.05.

## 3. Results

### 3.1. Phage CP30A Lysate is Generally Stable at Low and Ambient Temperatures

**Figure 1:**
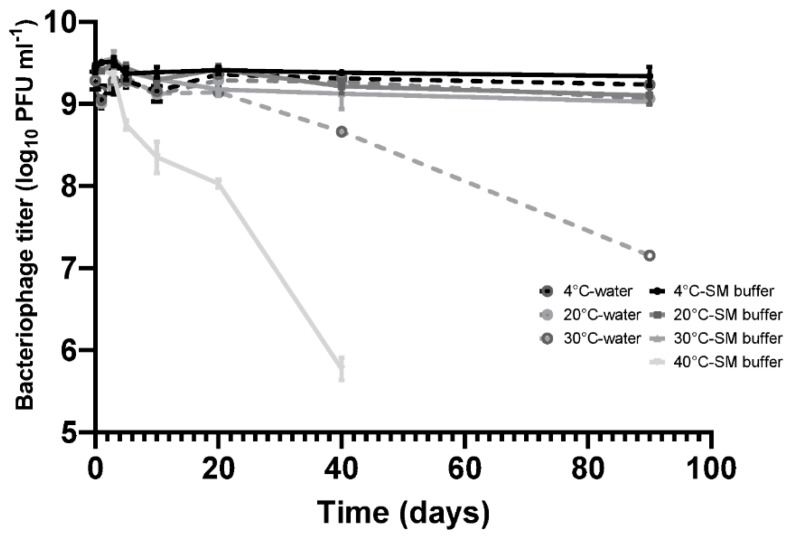
Stability of phage CP30A in RO (Reverse Osmosis) water and SM buffer (50 mM Tris.HCl (pH7.5), 100 mM NaCl, 8 mM MgSO4, 0.01% Gelatin). Samples were incubated in static incubators and aliquots were removed at each time point for phage titration. Error bars represent the standard deviation of the mean between three biological replicates.

In this study, bacteriophage CP30A was selected for phage lyophilization due to its ability to lyse a broad range of target bacteria, absence of host virulence associated genes and high efficiency in reducing levels of *C. jejuni* applied in chickens or in the laboratory [12,13,30]. Phage stability is one of the most important parameters for screens designed to establish therapeutic candidates, hence measuring stability of phage CP30A under different temperature conditions constituted the baseline for the study, which is shown in Figure 1.

Bacteriophage CP30A was stored statically at 4 °C for a 90-day period without any discernable fall in titer in either SM buffer or RO water. CP30A stored at 20 °C and 30 °C in SM buffer remained stable over 10 days of incubation with titer reductions recorded thereafter. Titer reductions of 0.33 ± 0.07 and 0.41 ± 0.07 log_10_ PFU mL^−1^ were observed at the 20 °C and 30 °C incubation temperatures, respectively, after 90 days. However, in comparison, the CP30A phage titer were less stable in RO water, notably at 30 °C where a reduction of 2.13 ± 0.07 log_10_ PFU mL^−1^ was observed at 90 days. Although improving stability, the use of SM buffer for incubation at 40 °C did not prevent titer losses of phage CP30A, which exhibited a significant decrease of 0.68 ± 0.09 log_10_ PFU mL^−1^ over 5 days and 3.66 ± 0.12 log_10_ PFU mL^−1^ after 40 days incubation. These results indicate phage CP30A is generally stable at low and ambient temperatures but is sensitive to long-term storage at temperatures >30 °C. Although phage CP30A maintained high stability in aqueous suspension, temperatures >30 °C are commonplace in many developing countries and it is therefore preferable to dehydrate the sample for preservation and transportation to minimize the chances of contamination and deliver reproducible titers.

### 3.2. Phage CP30A Supplemented with Tryptone Retained the Highest Titer Postlyophilization

**Figure 2:**
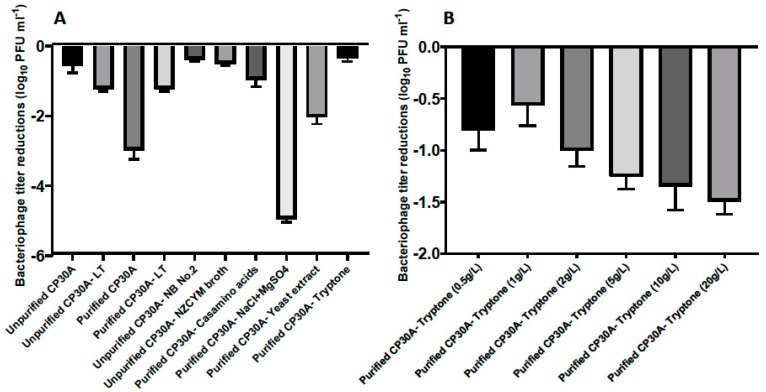
(**A**) Titer loss of lyophilized phage CP30A with different substances supplemented. The titer difference was calculated between phage initial stock and after rehydration and recorded as the titer loss. Individual excipients were added to purified phage CP30A to a final concentration of 1 g L^−1^ casamino acids, 5 g L^−1^ sodium chloride, 1 g L^−1^ magnesium sulfate, 5 g L^−1^ yeast extract and 1 g L^−1^ tryptone. (**B**) Titer loss of lyophilized phage with tryptone supplemented to different final concentrations. Error bars represent the standard deviation of the mean between three biological replicates.

To maintain CP30A phage titers, lyophilization was investigated using a Christ Alpha 1–2 LDplus freeze dryer to produce a dry phage-cake. The lyophilized product showed a typical moisture content of 13% after 24 hours of lyophilization, measured by TGA. The excipient formulation of leucine and trehalose described in a previous spray-drying study [26] showed a 1.22 ± 0.07 log_10_ PFU mL^−1^ titer reduction following lyophilization (Figure 2A). We further examined alternative excipients to maintain the phage titer more effectively during lyophilization.

The presence of bacterial debris could constitute a risk if applied to animals or edible goods, and therefore buffer exchange was required for filtered phage samples to make them feasible for biocontrol purposes. In this study, residual components of bacterial origin were removed from the phage samples by either pelleting phage by centrifugation, followed by resuspending pellet in reverse osmosis water to the same volume, or dialysis with spin column with a molecular weight cut-off of 100 kDa. Interestingly, phages harvested from the plate and filtered (unpurified CP30A with 15.7 mg mL^−1^ solid matter) exhibited a significantly lower titer reduction postlyophilization (2.43 log_10_ PFU mL^−1^) than CP30A phage preparations that had been buffer exchanged (purified CP30A, solid matter less than 0.1 mg mL^−1^ for both purification approaches). The solid content within the unpurified phage CP30A was mainly carried over from the culture medium. Therefore, we hypothesized that a medium ingredient may function or cofunction as an excipient to stabilize phage upon lyophilization. Centrifugation was adopted to efficiently and reproducibly remove non-phage debris from large-scale preparations.

Figure 2A illustrates that bacteriological agar was not a suitable stabilizing agent during lyophilization since there was no significant difference in phage titer changes between samples propagated using solid agar or liquid media methods. Ingredients from the NZCYM broth medium, including casamino acids, sodium chloride, magnesium sulfate, yeast extract and tryptone, were individually added to purified phage CP30A to determine their ability to stabilize phage upon lyophilization (Figure 2A). Among these, phage CP30A supplemented with tryptone to a final concentration of 1 g L^−1^ showed the least titer change caused by lyophilization (-0.35 ± 0.09 log_10_ PFU mL^−1^), which was similar to the titer loss we observed for unpurified phage CP30A (*p* > 0.05). Tryptone supplementations at a range of concentrations were analyzed to determine that 1 g L^−1^ tryptone offered the best protection to maintain phage titer during lyophilization (Figure 2B). Therefore, tryptone was applied to purified phage CP30A to a final concentration of 1 g L^−1^ throughout this study unless otherwise stated.

### 3.3. Storage and Rehydration of Lyophilized Phage CP30A

**Figure 3:**
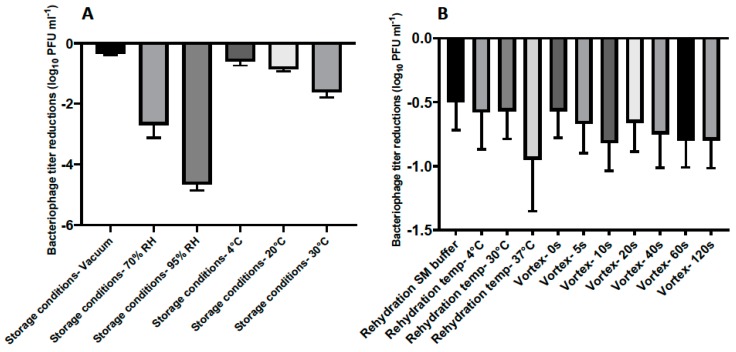
(**A**) Titer loss of lyophilized phage CP30A after incubation under different conditions for 7 days. (**B**) Titer loss of lyophilized phage CP30A reconstituted with different rehydration media and techniques. Samples were supplemented with tryptone to a final concentration of 1 g L^−1^, stored at 4 °C under vacuum and rehydrated at 4 °C in reverse osmosis water without vortex unless otherwise stated. Error bars represent the standard deviation of the mean between three biological replicates.

After optimizing the lyophilization formulation for phage CP30A, we then investigated if the reduced phage titer postlyophilization could be limited by applying different incubation conditions and rehydrating techniques (Figure 3). Lyophilized phages were stored at 4 °C under vacuum for one week and rehydrated using reverse osmosis water at 4 °C unless otherwise stated. 

The stability of lyophilized phage CP30A was negatively correlated with exposure to increasing relative humidity in a sealed glass vial after one week of storage, with the least titer reduction of 0.35 ± 0.03 log_10_ PFU mL^−1^ observed under vacuum (Figure 3A). Lyophilized phages stored at 4 °C and 20 °C showed similar levels of titer reduction (0.59 ± 0.14 and 0.85 ± 0.08 log_10_ PFU mL^−1^, respectively), which were significantly less than the titer loss observed in samples incubated at 30 °C. Compared to the storage conditions, the rehydration technique employed was of far lesser significance to product stability (Figure 3A and B). However, phages rehydrated at 37 °C with water exhibited a higher titer reduction of 0.95 ± 0.40 log_10_ PFU mL^−1^ than samples rehydrated at 4 °C and 20 °C in water (Figure 3B). No significant difference was observed between samples rehydrated with and without vortex and between samples rehydrated using water and SM buffer (*p* > 0.05). All the rehydrated phage preparations in this study retained the lytic profile of bacteriophage CP30A against susceptible *C. jejuni* and *C. coli* hosts [29].

### 3.4. Lyophilized Phage CP30A is Applicable for International Transportation

**Figure 4:**
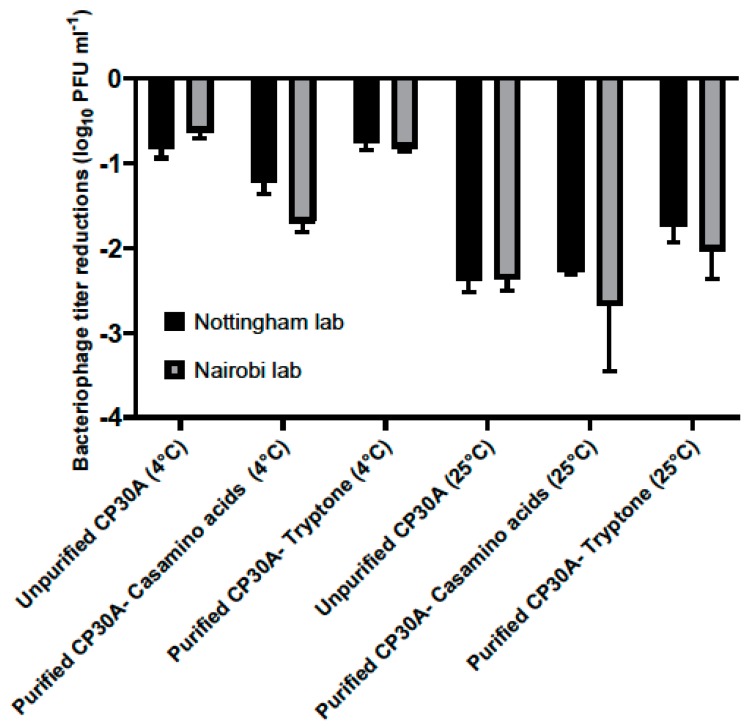
Titer loss of lyophilized phage CP30A after international transportation. Two identical sets of phage samples were aliquoted from the same stock. After lyophilization, one set was shipped to Kenya with and without temperature control and a second set was incubated in Nottingham lab to simulate the transport temperatures as a control set. Error bars represent the standard deviation of the mean between three biological replicates.

Lyophilized phage CP30A was shipped from Nottingham, UK to Nairobi, Kenya to assess the stability of the preparation under the conditions associated with international travel. The product travelled a straight-line distance of 6987 km that took 23 days. Phage CP30A was shipped under vacuum refrigeration at 4 °C or without temperature control and exposure to ambient temperatures. The shipment samples contained purified phage supplemented with casamino acid, tryptone and unpurified phage. A phage control group was set up in the Nottingham laboratory and stored at either 4 °C or 25 °C to simulate the chilled and ordinary shipping services. Upon arrival in Nairobi, lyophilized phage CP30A was reconstituted at 4 °C and subsequently tittered. The control samples were rehydrated and enumerated in Nottingham lab on the same day (Figure 4).

For the lyophilized unpurified phage CP30A preparation, titer reductions of 0.81 ± 0.12 log_10_ PFU mL^−1^ and 0.63 ± 0.08 log_10_ PFU mL^−1^ was observed for the samples stored at 4 °C in Nottingham lab and samples shipped chilled to Nairobi, respectively. Supplementing purified phage CP30A with tryptone revealed similar results with titer losses of 0.76 ± 0.07 log_10_ PFU mL^−1^ and 0.81 ± 0.44 log_10_ PFU mL^−1^ for the control samples stored at 4 °C and the samples shipped under refrigerated conditions, respectively. The greatest titer change was observed for lyophilized phage CP30A with casamino acids added, which was consistent with the formulation observations. Rehydrated phage CP30A was incubated at 4 °C for a further month without any further fall in titer. Lyophilized phage samples shipped under room conditions or incubated at 25 °C showed a higher titer loss compared with the samples that were maintained chilled, suggesting the lyophilized phages are temperature sensitive.

### 3.5. Capsid Integrity Postlyophilization

**Figure 5:**
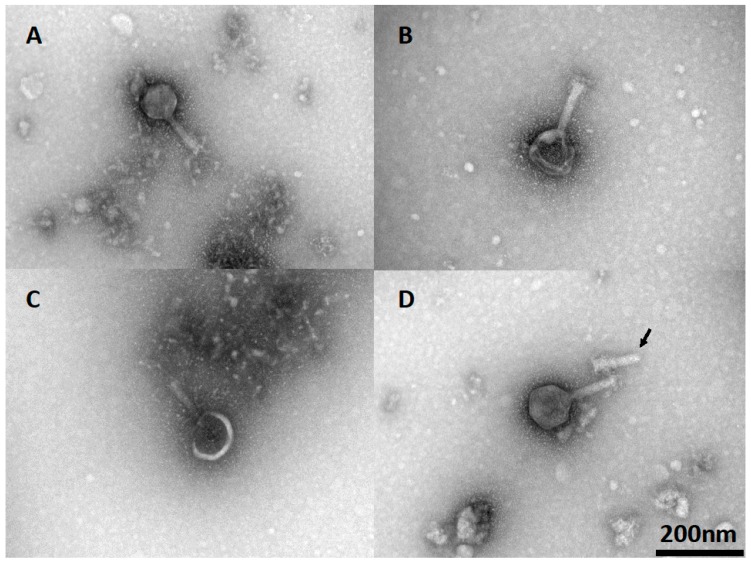
TEM of lyophilized phage CP30A after rehydration. (**A**) Wild-type phage CP30A. (**B**) Phage CP30A with a deformed capsid. (**C**) Phage CP30A with a broken capsid. (**D**) Phage CP30A capsid detached from its tail (arrowhead). The scale bar of 200 nm in panel D is applicable to all panels.

Phage viability and the efficiency of plating (EOP) are dependent on the integrity of the phage particles. The morphology of phage CP30A, together with lyophilized and rehydrated samples, was determined via TEM to investigate any potential cause for the reductions in titer observed postlyophilization. Wild-type phage CP30A had a typical icosahedral head and contractile tail as shown in Figure 5A. After lyophilization and reconstitution, 48% of visualized phage (*n* = 50) showed either deformed, damaged or detached capsid (Figure 5B–D). Notably, phage aggregation was not observed for any of the visualized fields.

### 3.6. Tryptone Forms an Amorphous Network to Protect Phage During Lyophilization

**Figure 6:**
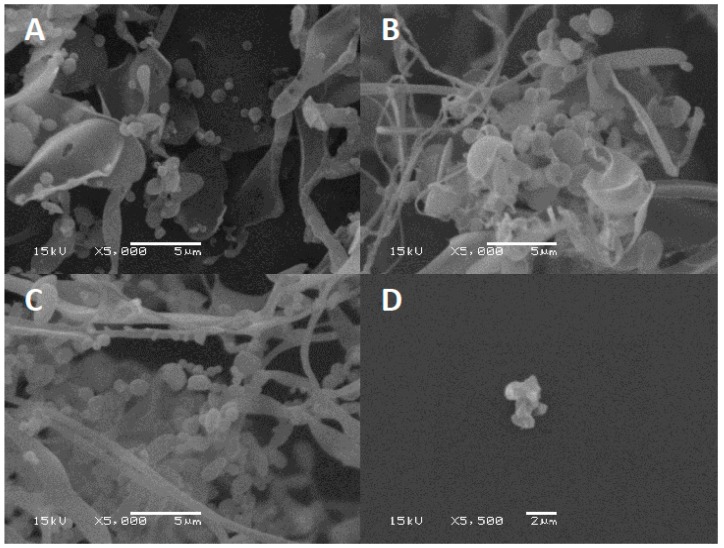
SEM of lyophilized phage CP30A. (**A**) Phage CP30A with tryptone imaged immediately after lyophilization. (**B**) Lyophilized phage CP30A with tryptone and incubated at 4 °C for 23 days. (**C**) Lyophilized phage CP30A with tryptone and incubated at 25 °C for 23 days. (**D**) Lyophilized phage CP30A without any addition. The scale bars are indicated in situ for each panel.

Excipients play a key role in preventing phages from inactivation during the drying process by forming a protective amorphous layer. Lyophilized phage CP30A was examined using SEM to examine the structures in which the phages were embedded. After lyophilization, tryptone formed an amorphous branched network of sheets on which were located sphere-like shells (d = 1079.5 ± 140 nm) (Figure 6A). In contrast, lyophilized CP30A phage in the absence of the excipient formed aggregated structures (Figure 6D). After 23 days incubation at 4 °C and 25 °C, SEM revealed that the protective layer remained intact, appearing similar to that observed immediately after lyophilization.

## 4. Discussion

As an alternative approach to control bacterial pathogens from various reservoirs, bacteriophages have been a focus for research in recent years. A role for which phages are well suited is as specific antimicrobial agents for food safety and farm animal production [31]. The scale of production and transport for these applications requires a move to industrialization. There are some notable successes in bringing phages to the food sector, but to expand these possibilities will require the expense of high-volume bulk transport be negated. A straightforward way to lower shipping cost and storage volume is to dehydrate phages. Studies have shown both lyophilization and spray-drying process can effectively produce phage powder/cake without substantially impairing phage viability [23,25,32,33].

The objective of the present study was to design a systematic lyophilization approach for *Campylobacter* bacteriophage that is feasible for transportation to enable phage cocktail candidates to be shared in the pursuit of the international development of phage products for regional applications. Phage CP30A was used in the study as an exemplar of a virulent broad-host phage with robust characteristics that make it suitable for biocontrol applications [13]. Indeed, we demonstrate that CP30A was remarkably stable when stored in a simple buffered aqueous suspension, showing no significant loss in titer when stored at 4 °C for 90 days or low titer reductions of <0.5 log_10_ PFU mL^−1^ at 20 °C and 30 °C. Unlike studies which seek to use lyophilization to stabilize phages for long-term storage [34], we sought to be able distribute lyophilized phages with minimal titer loss for immediate research applications, and in this context the reconstituted phage retained their host lysis profiles.

During lyophilization, the presence of excipients is critical to form amorphous structures to protect phage and retain viability [35]. Sugars are generally considered as good candidates for use as phage excipients [36]. We have investigated the formulation of leucine and trehalose to lyophilize CP30A phage, which showed a 1.22 log_10_ PFU mL^−1^ titer drop postlyophilization. Although this titer reduction was significantly less than that of a spray-drying procedure using a similar formulation [26], we aimed to further reduce the fall in titer by analyzing other suitable excipients. Among the substances tested, tryptone and casamino acids additions showed the best performance in terms of stabilizing phages from inactivation during lyophilization.

TEM images of rehydrated phage CP30A suggested that the titer loss during lyophilization was caused by broken, deformed, or detached capsid structures compared with the morphology of untreated phage, an observation that could be related to the formation of ice crystals. The successful rehydration of phage preparations with low titer losses demonstrated that the phage structures remained intact and viable. Frequently, *C. jejuni* phages can irreversibly form aggregates at high titers [13]. A significant benefit of the lyophilization procedure was that this did not occur at reconstitution.

Vandennheuvel’s group have reported spray-dried phages stored at 4 °C and 0% relative humidity were significantly more stable than samples stored at 25 °C and 0% relative humidity [37]. We also observed reduced viability for lyophilized CP30A phage samples stored in vacuo at 20 °C and 30 °C. However, SEM images of the lyophilized structures showed no observable differences in terms of the amorphous structures present for lyophilized CP30A phage preparations stored at higher temperatures with those stored under refrigerated conditions. The higher temperatures may cause recrystallization of the amorphous shell, which may exacerbate thermal instability of the phages embedded in the amorphous shell. Lyophilized CP30A phage was significantly more stable under vacuum than under high humidity conditions, which would consequently lead to an increase in moisture content. This is consistent with the negative correlation between the moisture content of lyophilized phage cake and the phage lytic activity reported by Puapermpoonsiri et al. [23]. Powder rehydration properties are important factors in biopharmaceutical formulation. Cox et al. pointed out a slow rehydration resulted in a significant higher titer for phages after lyophilization [38]. Therefore, the relationship between rehydration media, techniques and the resulting phage titer were analyzed here. We found a rehydration temperature of 37 °C to have minimal effect, lower temperatures to have no observable effect and agitation in the form of vortex during rehydration to have no significant effect on the titer recovered. Rehydration using SM buffer and reverse osmosis water showed similar titer loss (*p* > 0.05), suggesting the osmotic shock damage to phages during lyophilization described by Shapira and Kohn was not evident here [39]. These results helped us understand the titer reduction for phage CP30A was mainly from the lyophilization process and storage, whereas rehydration process has a minor impact.

International shipment for lyophilized CP30A phage was arranged to verify that the formulation was suitable for long-distance transport and immediate use at delivery. Lyophilized samples were more stable in cold-chain transportation than ambient shipping regardless of the excipients used. The excipient tryptone conferred the greatest stability to lyophilized CP30A phages. The low titer loss using tryptone was similar to that of the unpurified phage CP30A lysate, suggesting tryptone is the functional excipient carried over from the host culture medium. Although tryptone is not approved as an inactive ingredient by the FDA, considering it is the digestion products of casein, it could be an acceptable excipient for a food and pharmaceuticals.

To our knowledge, this is the first study to report the stability of lyophilized *Campylobacter* phage and where the small titer loss upon lyophilization is favorable compared to other studies of bacteria-specific phages [34]. Future work could explore alternative excipients, especially the formulation of trileucine and pullulan that have enhanced the performance of the spray-drying technique to stabilize phage titers [26]. Further study could also focus on the application of secondary drying at warmer temperatures to desorb any bound water present after primary drying to reduce the residual moisture content. Although Puapermpoonsiri et al. suggested an optimal moisture content of between 4% and 6% for lyophilized phages in their study [23], the optimal range could vary between phages. Future improvements should establish the optimal moisture content for phage CP30A to use this for reference as new *Campylobacter* phage cocktail candidates are discovered. In summary, lyophilization can produce stable cake of CP30A phage with a titer loss of less than 1 log_10_ PFU mL^−1^ after international transportation and is suitable for batch phage preparation when phage titers need to be assured for immediate use.
